# A PNPLA3–NACC1–RIPK3 pathway mediates macrophage necroptosis and inflammation in MASLD

**DOI:** 10.1097/HC9.0000000000000890

**Published:** 2026-01-21

**Authors:** Xinjia Wang, Lu Bian, Zhuoying Feng, Kyle O’Shaughnessy, Andrew C. Kwong, Eun Hee Ha, Lei Wang, Weibo Chen, Xianfang Wu

**Affiliations:** 1 Department of Microbial Science in Health, Cleveland Clinic, Cleveland, Ohio, USA; 2 Department of Genetics and Genome Sciences, School of Medicine, Case Western Reserve University, Cleveland, Ohio, USA; 3 The Biological and Biomedical Sciences Program, Harvard Medical School, Boston, Massachusetts, USA; 4 Cleveland Clinic College of Medicine at Case Western Reserve University, Cleveland, Ohio, USA

**Keywords:** genetic disease, MASLD, multicellular liver culture, NACC1 inhibition, necroptosis, NF-kB activation

## Abstract

**Background:**

The 148M variant of PNPLA3 is a major genetic risk factor for metabolic dysfunction–associated steatotic liver disease (MASLD), yet its macrophage-specific role remains unclear. We investigated how PNPLA3-148M alters macrophage behavior and multicellular liver pathology under lipotoxic stress.

**Methods:**

We used a human induced pluripotent stem cell (iPSC)-derived multicellular liver culture comprising hepatocytes, hepatic stellate cells (HSCs), and isogenic macrophages that differed only at PNPLA3 (148M vs. 148I). Cultures were exposed to lipotoxic conditions to induce MASLD. We quantified inflammatory cytokines, oxidative stress, hepatocyte lipid accumulation, and HSC activation. Macrophage death pathways were profiled (apoptosis, pyroptosis, necroptosis), with emphasis on RIPK3 expression and phosphorylation. Downstream effects on hepatocytes and HSCs were assessed.

**Results:**

Under lipotoxic stress, 148M macrophages amplified MASLD-like features. *PNPLA3* transcripts were induced in macrophages—rising in iPSC-derived macrophages from lipotoxic cultures, and in Kupffer cells isolated from murine MASLD. Single-cell RNA-seq further confirmed *PNPLA3* expression in human liver macrophage clusters, in addition to hepatocytes and HSCs. Although *PNPLA3* mRNA was comparable between genotypes, 148M macrophages displayed higher PNPLA3 protein and increased necroptosis, evidenced by elevated RIPK3 expression and phosphorylation without changes in apoptosis or pyroptosis. Integrative analyses identified NACC1 as a key transcriptional regulator of *RIPK3*, with NF-κB–linked upregulation of NACC1 in 148M macrophages. NACC1 inhibition (genetic or NIC3) reduced RIPK3, suppressed necroptosis, and lowered pro-inflammatory cytokine secretion. NIC3 additionally decreased hepatocyte lipid accumulation and ATP and diminished HSC activation markers.

**Conclusions:**

The PNPLA3 148M variant promotes MASLD through a macrophage-specific NF-κB–NACC1–RIPK3 axis that enhances necroptosis and inflammatory signaling, thereby exacerbating hepatocyte steatosis and HSC activation. NACC1 emerges as a tractable therapeutic target for genetically at-risk individuals.

## Graphical abstract

**Figure:**
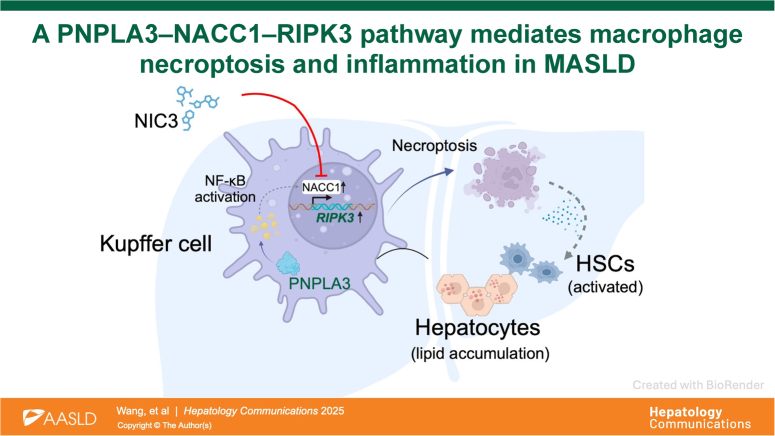

## INTRODUCTION

Patatin-like phospholipase domain-containing protein 3 (*PNPLA3*), also known as adiponutrin, is a lipid droplet–associated protein encoded by the *PNPLA3* gene on chromosome 22. PNPLA3 is highly expressed in hepatocytes, hepatic stellate cells (HSCs), and adipocytes, where it localizes to the cytosolic face of lipid droplets and regulates lipid remodeling through triglyceride lipase and acyltransferase activities.^1^ The common I148M variant (rs738409 C>G, referred to as 148M in contrast to the wild-type 148I) results in a substitution of isoleucine with methionine at residue 148 within the catalytic domain. This substitution abolishes its triglyceride hydrolase activity, causing lipid droplet accumulation.^2^ The 148M variant represents one of the strongest genetic risk factors for metabolic dysfunction–associated steatotic liver disease (MASLD) and its inflammatory and fibrotic progression; however, the cellular and molecular mechanisms underlying this association remain only partially defined.^3^

While most prior studies have focused on the impact of PNPLA3-148M in hepatocytes and HSCs, its potential role in macrophages remains largely unexplored. Given that macrophages are key mediators of hepatic inflammation and cell death in MASLD, we hypothesized that the PNPLA3-148M variant could alter macrophage lipid handling and stress responses. To test this, we employed a lipotoxic liver culture model,^4^^–^^6^ where the crosstalk between hepatocytes, stellate cells, and macrophages recapitulates the inflammatory microenvironment of MASLD. Our findings reveal a macrophage-intrinsic, PNPLA3-dependent mechanism that amplifies inflammatory and fibrotic signaling in this context. Collectively, this work broadens the functional scope of PNPLA3 beyond hepatocytes and HSCs and identifies NACC1 as a promising therapeutic target for both genetically at-risk and non-risk individuals.

## METHODS

### Stem cell maintenance and editing

The isogenic pair of PNPLA3^148I^ and PNPLA3^148M^ cells was generated using CRISPR Cas9-mediated gene editing, as described previously.^4^ Correct clones were characterized for their growth rate, pluripotent marker expression, and trilineage differentiation capacity.

### Establishment of a multicellular liver culture

Human-induced pluripotent stem cell (hiPSC)-derived hepatocytes, HSCs, and macrophages were differentiated separately and assembled into a coculture as described previously.^4^^–^^6^ Cocultures were maintained in a basal maintenance medium (BMM) that consisted of glucose-free DMEM supplemented with 5% knockout serum replacement (KOSR), 2% B-27, 3 U/mL heparin, 200 μg/mL transferrin, 30 ng/mL EGF, 5 μM retinol, and 0.5 μM dexamethasone. The 12-well plate containing cocultures was placed on an orbital shaker platform at a speed of 40 rpm/min, and medium was replenished with fresh medium every 2 days or as indicated in the figure legend. For healthy or lipotoxic conditions, BMM was supplemented with either insulin (0.7 nM) and glucose (6.0 mM) or insulin (7.0 nM), glucose (25.0 mM), and free fatty acid (oleic acid, 68 μM and palmitic acid, 45 μM).

### Human monocyte isolation and macrophage differentiation

Human peripheral blood mononuclear cells (PBMCs, catalog number: 70025) were obtained from Stemcell Technologies. Monocytes were isolated by negative selection using the Pan Monocyte Isolation Kit (Miltenyi Biotec) following the manufacturer’s instructions. Purity was assessed by flow cytometry using anti-CD14 and anti-CD3 antibodies; post-isolation cells were CD3^−^CD14^+^ with >97% purity in all preparations.

For macrophage differentiation, purified monocytes were cultured in RPMI 1640 supplemented with 10% FBS, 1% penicillin–streptomycin, 2 mM L-glutamine, and recombinant human M-CSF (20 ng/mL; PeproTech) for 6 days.

### Single-cell RNA-seq analysis

Single-cell RNA sequencing (scRNA-seq) data were obtained from the Human Liver Cell Atlas, specifically from GEO accession GSE192742,^7^ in the form of raw count matrices and accompanying cell metadata. All downstream analysis was conducted in R (v4.4.1) using the Seurat package (v5.3.0). To prepare the data for visualization and interpretation, we applied the standard Seurat pipeline, including NormalizeData, FindVariableFeatures, and ScaleData functions. As the original study had already removed low-quality cells and doublets, we retained the provided cell annotations for downstream analyses and UMAP visualization. Expression distribution across major liver cell populations was visualized using the VlnPlot function in Seurat.

### Statistical analysis

Detailed information regarding all statistical tests, the specific value of ‘n’ and its representation (eg, number of cell clones or experimental replicates) can be found in the respective figure legends. Graphs depict results presented as means ± SD. Group comparisons were conducted using the unpaired *t* test with the Welch correction or one-way ANOVA/Tukey post-hoc test, as appropriate, to calculate precise *p*-values unless otherwise specified. Statistical analyses were carried out using GraphPad Prism 10. All *p*-values are reported, and a *p*-value >0.05 denotes statistical non-significance. Additional detailed information regarding the materials and methods is available in the Supplementary Data, http://links.lww.com/HC9/C235.

## RESULTS

### Macrophage-specific PNPLA3-148M variant promotes inflammatory and fibrotic phenotypes

**FIGURE 1:**
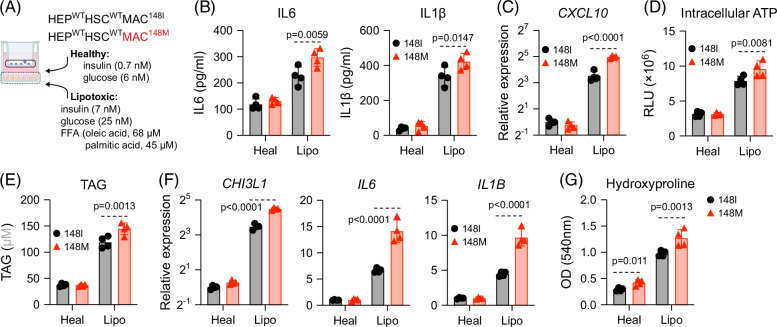
Macrophage-specific PNPLA3-148M variant promotes inflammatory and fibrotic phenotypes. (A) An isogenic pair of human iPSC-derived liver cultures, differing only in *PNPLA3* genotype of the macrophages, was maintained in either a healthy or lipotoxic medium. (B) At 2 weeks post-exposure, supernatants were collected for analysis of cytokines by ELISA. (C–E) From the experiments described in (B), hepatocytes were purified for analysis of *CXCL10* transcript by RT-qPCR (C), intracellular ATP (D), and triacylglycerol levels (E). (F) From the experiments described in (B), macrophages were purified for analysis of inflammatory gene transcripts by RT-qPCR. (G) From the experiments described in (B), HSCs were purified for analysis of collagen levels by the hydroxyproline assay. Shown are the means ± SD from 4 independent experiments. Statistical analysis was performed using unpaired *t* tests with Welch correction or one-way ANOVA/Tukey post-hoc test to calculate *p*-values. Abbreviations: HSCs, hepatic stellate cells; iPSC, induced pluripotent stem cell; RT-qPCR, reverse transcription–quantitative polymerase chain reaction; WT, wild type.

To investigate the potential role of macrophage PNPLA3 in MASLD pathogenesis, we utilized our previously established isogenic human iPSC-derived liver culture system.^4^^–^^6^ This model cocultures human iPSC-derived hepatocytes, HSCs, and macrophages at an 8:1:1 ratio, approximating the cellular composition of a healthy liver. For this study, we generated 2 otherwise identical liver cultures differing only in the *PNPLA3* genotype of the macrophages—one carrying the 148I allele and the other the 148M variant (Figure 1A).

Both liver cultures were exposed to a lipotoxic medium, a condition we previously demonstrated to induce MASLD-like pathology.^4^^,^^6^^,^^8^^,^^9^ After 2 weeks, we assessed inflammatory cytokines and found that IL6 and IL-1β were elevated in cultures harboring 148M macrophages compared with those with 148I macrophages, where cytokine levels were similar under healthy conditions (Figure 1B).

Prompted by this finding, we further examined MASLD-associated phenotypes across different liver cell types. Hepatocytes from cultures containing 148M macrophages displayed increased *CXCL10* and ATP levels (Figure 1C-D)—both markers of oxidative stress in MASLD,^10^^,^^11^ and accumulated more lipids than hepatocytes from 148I cultures (Figure 1E). Again, these differences were not seen under healthy conditions.

We next evaluated macrophages and HSCs from the 2 culture systems. Under lipotoxic conditions, both macrophage genotypes showed increased reactive oxygen species (ROS)—a well-documented feature in MASLD—with the macrophages carrying 148M variant displaying modestly higher ROS (Supplemental Figures S1A, B, http://links.lww.com/HC9/C235) and expressed higher transcript levels of *CHI3L1*, *IL1B*, and *IL6* (Figure 1F). HSCs from cultures with 148M macrophages also had higher hydroxyproline content, indicating greater HSC activation (Figure 1G). Longitudinal monitoring of cell viability over the 2-week treatment period revealed no major differences, aside from a slight decline in 148M macrophage viability late in treatment (Supplemental Figure S1C, http://links.lww.com/HC9/C235). Finally, we included 2 additional isogenic pairs of liver cultures, derived from different iPSC 148I and 148M clones, and observed a similar trend, with stronger inflammatory and fibrotic responses in cultures harboring 148M macrophages (Supplemental Figures S1D–G, http://links.lww.com/HC9/C235).

Together, these findings reveal that macrophages carrying the PNPLA3 148M variant exacerbate lipotoxic liver injury, highlighting a previously unrecognized role for macrophage PNPLA3 in driving MASLD-like pathology.

### PNPLA3 is upregulated in macrophages under lipotoxic stress

**FIGURE 2:**
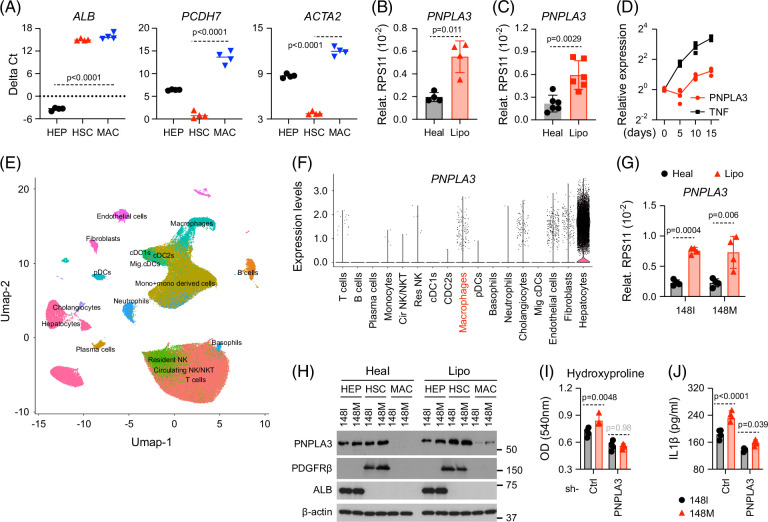
PNPLA3 is upregulated in macrophages under lipotoxic stress. (A) Human iPSC-derived liver cultures (all 148I cells) were exposed to lipotoxic conditions for 2 weeks before cells were purified for analysis of transcript levels of cell type-specific marker genes by RT-qPCR. (B) Human iPSC-derived macrophages were exposed to conditioned medium collected from liver cultures for 5 days before being analyzed for *PNPLA3* transcript by RT-qPCR. (C) Primary human Kupffer cells from 3 different donors were exposed to conditioned medium collected from liver cultures for 5 days before being analyzed for *PNPLA3* transcript by RT-qPCR, with technical duplicates for each donor. (D) Liver cultures were exposed to lipotoxic medium for 15 days, and macrophages were collected at the indicated time points for analysis by RT-qPCR. (E, F) UMAP plot showing subclustering of different cell types from human liver samples (E, GSE192742), and expression distribution of *PNPLA3* across major liver cell populations was shown (F). (G) Liver cultures were assembled as described in Figure 1A. Macrophages were harvested for analysis of *PNPLA3* transcript by RT-qPCR. (H) Liver cultures harboring all *PNPLA3* 148I or 148M cells were exposed to the indicated culture medium for 2 weeks. Hepatocytes (HEP), HSCs, and macrophages (MAC) were then purified for western blot analysis. (I, J) Liver cultures were assembled as described in Figure 1A, except for macrophages being transduced with control shRNA or *PNPLA3* shRNA. Liver cultures were exposed to lipotoxic medium for 2 weeks before HSCs were purified for analysis of hydroxyproline levels (I) and supernatants for IL-1β levels (J). Shown are the means ± SD from n=4 independent experiments. Statistical analysis was performed using unpaired *t* tests with Welch correction or one-way ANOVA/Tukey post-hoc test to calculate *p*-values. Abbreviations: HSCs, hepatic stellate cells; iPSC, induced pluripotent stem cell; RT-qPCR, reverse transcription–quantitative polymerase chain reaction; UMAP, Uniform Manifold Approximation and Projection.

To investigate the mechanisms underlying enhanced effects mediated by PNPLA3 148M in macrophages, we first confirmed that our iPSC-derived macrophages were not contaminated by hepatocytes or HSCs, which express high levels of PNPLA3. They showed negligible or undetectable levels of *ALB*, *PCDH7*, and *ACTA2* (Figure 2A), established markers of hepatocytes and HSCs, respectively.

These findings support that the heightened susceptibility of liver cultures containing 148M macrophages stems from PNPLA3 within macrophages. Indeed, *PNPLA3* transcripts were upregulated in iPSC-derived macrophages under lipotoxic conditions (Figure 2B). Similar upregulation was observed in primary human Kupffer cells (KCs) and monocyte-derived macrophages exposed to conditioned medium from lipotoxic liver cultures (Figure 2C and Supplemental Figures S2A–D, http://links.lww.com/HC9/C235), as well as in liver macrophages from a murine MASLD model (Supplemental Figure S2E, http://links.lww.com/HC9/C235). Interestingly, *PNPLA3* upregulation occurred mainly at later stages of lipotoxic exposure, whereas TNF increased steadily throughout treatment (Figure 2D) and was correlated with disease progression in patients,^12^ suggesting distinct regulatory kinetics. scRNA-sequencing of human livers further confirmed *PNPLA3* expression in liver macrophages, in addition to hepatocytes and HSCs (Figures 2E, F). Notably, *PNPLA3* upregulation in hepatocytes and HSCs was also observed in both our model and the murine MASLD model (Supplemental Figures S2F, G, http://links.lww.com/HC9/C235), consistent with MASLD patient livers (Supplemental ​​​​ Figure S2H, http://links.lww.com/HC9/C235).

We next compared *PNPLA3* expression between genotypes. Although mRNA levels were similar in 148I and 148M macrophages (Figure 2G), protein levels were consistently higher in 148M cells (Figure 2H), a trend reproducible across 2 independent iPSC pairs (Supplemental Figures S2I, J, http://links.lww.com/HC9/C235). This finding suggests that the 148M variant does not increase *PNPLA3* transcription or mRNA stability, consistent with previous observations in hepatocytes and HSCs.^4^^,^^13^

To test whether elevated PNPLA3 protein drives the heightened response, we knocked down PNPLA3 in both genotypes. Under lipotoxic conditions, shRNA normalized PNPLA3 protein levels between 148I and 148M macrophages (Supplemental Figure S2K, http://links.lww.com/HC9/C235), which reduced HSC activation and inflammatory signaling and improved liver phenotypes (Figures 2I, J). These findings align with a recent phase I clinical trial demonstrating the benefits of *PNPLA3* silencing using antisense oligonucleotides.^14^

Collectively, these results indicate that the enhanced vulnerability conferred by 148M macrophages is primarily due to elevated PNPLA3 protein levels in these cells.

### SREBF1 regulates PNPLA3 induction in macrophages

To understand how PNPLA3 expression is regulated in macrophages under lipotoxic stress, we examined transcriptional pathways involved in lipid metabolism. PNPLA3 is a lipid droplet–associated enzyme with roles in lipid remodeling^15^ and is strongly induced by elevated levels of insulin, glucose, and free fatty acids.^16^^,^^17^ These cues activate the SREBP (sterol regulatory element-binding protein) pathway, a central regulator of lipid synthesis.^18^ Among the SREBP family, SREBF1 and SREBF2 control fatty acid and cholesterol metabolism, respectively, prompting us to test their roles in PNPLA3 regulation in macrophages.

**FIGURE 3:**
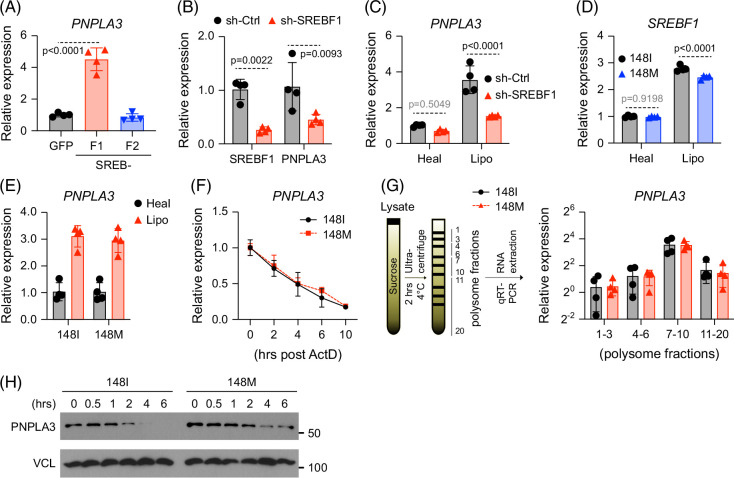
SREBF1 regulates PNPLA3 induction in macrophages. (A) Human iPSC-derived macrophages were transduced with lentiviral vectors expressing GFP, SREBF1, or SREBF2, before being collected for analysis of *PNPLA3* transcripts. (B) Human iPSC-derived macrophages were transduced with control shRNA or *SREBF1* shRNA before being collected for analysis by RT-qPCR. (C) Human iPSC-derived macrophages were transduced with either control shRNA or *SREBF1* shRNA, exposed to conditioned medium collected from liver cultures for 5 days, and analyzed for *PNPLA3* transcript by RT-qPCR. (D) Human iPSC-derived macrophages harboring either 148I or 148M variant were exposed to conditioned medium collected from liver cultures for 5 days before being analyzed for *SREBF1* transcripts by RT-qPCR. (E, F) Liver cultures were assembled as described in Figure 1A, and macrophages were analyzed for *PNPLA3* transcript by RT-qPCR (E). Macrophages were treated with actinomycin D (2.0 μg/mL) for the indicated period of time before being analyzed for *PNPLA3* transcripts by RT-qPCR. Values were normalized to *t*=0. (G) Liver cultures were assembled as described in Figure 1A, and macrophages were treated with cycloheximide (50 µg/mL) before being subject to polysome profiling. PNPLA3 transcript distribution was quantified by RT-qPCR and expressed as fold changes relative to fractions 1–3. (H) Liver cultures were assembled as described in Figure 1A, and macrophages were treated with cycloheximide (50 µg/mL) for the indicated period of time before being analyzed by western blot. Shown are the means ± SD from n=4 independent experiments. Statistical analysis was performed using unpaired *t* tests with the Welch correction or one-way ANOVA/Tukey post-hoc test to calculate exact *p* values. Abbreviations: iPSC, induced pluripotent stem cell; RT-qPCR, reverse transcription–quantitative polymerase chain reaction; SREBF1/2, sterol regulatory element-binding transcription factor 1/2.

Overexpression studies showed that SREBF1, but not SREBF2, increased *PNPLA3* transcript levels in human iPSC-derived macrophages (Figure 3A). Correspondingly, *SREBF1* knockdown reduced *PNPLA3* expression under both healthy and lipotoxic conditions (Figures 3B, C), indicating that SREBF1 is essential for PNPLA3 expression in macrophages. Lipotoxic medium also robustly induced *SREBF1* expression in both genotypes, although to a slightly lesser extent in 148M macrophages (Figure 3D).

Because *PNPLA3* mRNA levels were similar between genotypes, we next sought to understand the increased PNPLA3 protein in 148M macrophages. Actinomycin D chase assays showed comparable transcript stability in both genotypes, with an estimated half-life of 4 hours (Figures 3E, F). Polysome profiling revealed similar ribosome loading on *PNPLA3* mRNA (Figure 3G), indicating comparable translation efficiency. In contrast, cycloheximide-chase experiments demonstrated that PNPLA3-148M protein is more stable than the 148I variant (Figure 3H). Together, these results indicate that the elevated PNPLA3 protein in 148M macrophages is due primarily to enhanced protein stability, not differences in transcription or translation.

### 148M macrophages exhibit enhanced lipid accumulation, LDH release, and necroptosis, independent of apoptosis or pyroptosis

**FIGURE 4:**
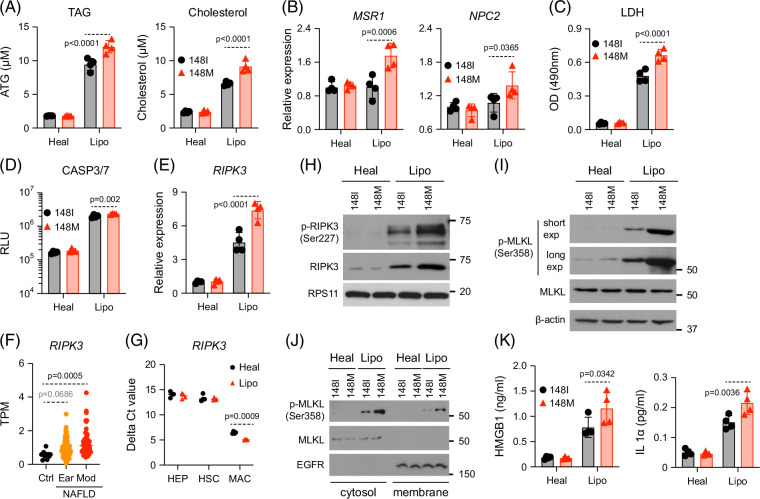
148M macrophages exhibit enhanced lipid accumulation, LDH release, and necroptosis, independent of apoptosis or pyroptosis. Liver cultures harboring either 148I or 148M macrophages were exposed to healthy or lipotoxic medium for 2 weeks. (A, B) Macrophages were collected for analysis of intracellular triacylglyceride (TAG) and cholesterol levels (A), and for analysis by RT-qPCR (B). (C) Macrophages were harvested, washed 3 times with the corresponding fresh medium, and then maintained in fresh medium for 48 hours. Supernatants were analyzed for LDH levels. (D, E). Macrophages were harvested for analysis of caspase 3/7 activity (D) or for analysis of *RIPK3* transcript levels (E). (F) *RIPK3* transcripts were analyzed using transcriptomic data from MASLD patients (GSE135251). Patients were divided according to their disease stage: early (Ear) or moderate (Mod). (G) Cells were purified for analysis of *RIPK3* transcript (normalized to housekeeping gene *RPS11*). (H, I) Macrophages were harvested for western blot analysis. (J). Macrophages were subject to subcellular protein fractionation and analyzed by western blot. EGFR was used as a marker for a membrane-bound protein. (K) Macrophages were harvested, washed with the corresponding fresh medium, and then maintained in fresh medium for 4 days. Supernatants were analyzed by ELISA. Shown are the means ± SD from n=4 independent experiments. Statistical analysis was performed using unpaired *t* tests with Welch correction or one-way ANOVA/Tukey post-hoc test to calculate exact *p* values. Abbreviations: EGFR, epidermal growth factor receptor; HEP, hepatocytes; HSC, hepatic stellate cell; LDH, lactate dehydrogenase; MAC, macrophages; MASLD, metabolic dysfunction–associated steatotic liver disease; RT-qPCR, reverse transcription–quantitative polymerase chain reaction.

Given the differential response of the 2 *PNPLA3* genotypes under lipotoxic stress (Figures 1, 2), we next investigated the underlying mechanisms. Lipotoxic medium increased intracellular lipid levels in both macrophage types, with a greater accumulation in 148M macrophages (Figure 4A and Supplemental Figure S3A, http://links.lww.com/HC9/C235). This was accompanied by higher transcript levels of *MSR1* and *NPC2* (Figure 4B), genes involved in lipid uptake and cholesterol trafficking, respectively, suggesting altered lipid handling in 148M cells.

Lipid overload is a key driver of MASLD and can trigger lipotoxic stress and cell death.^19^ Consistent with an adaptive response, we observed *APOE* upregulation (Supplemental Figure S3B, http://links.lww.com/HC9/C235), a mediator of lipid efflux.^20^ To assess cell damage, we measured the release of lactate dehydrogenase (LDH), a marker of membrane integrity loss.^21^ Lipotoxic treatment increased LDH release in both genotypes, but was higher in 148M macrophages (Figure 4C), indicating greater membrane damage and cell death.

To define the mode of cell death, we evaluated 3 major pathways: apoptosis, pyroptosis, and necroptosis. Apoptosis appeared comparable between the 2 genotypes, as evidenced by similar levels of caspase 3/7 activity (Figure 4D) and cleaved PARP (Supplemental Figures S3C–E, http://links.lww.com/HC9/C235). Pyroptosis also did not differ significantly, with both genotypes showing similar induction of inflammasome-related genes (Supplemental Figure S3F, http://links.lww.com/HC9/C235) and comparable caspase-1 activity (Supplemental Figure S3G, http://links.lww.com/HC9/C235), the canonical effector of inflammasome-mediated pyroptosis.

In contrast, we observed a clear difference in necroptosis. Lipotoxic medium induced *RIPK1* and *RIPK3*, though not *MLKL* (Figure 4E and Supplemental Figure S3H, http://links.lww.com/HC9/C235), in both genotypes—a pattern consistent with transcriptomic data from MASLD patients (Figure 4F).^22^ Importantly, *RIPK3* expression was higher in 148M macrophages (Figure 4E). Notably, macrophages expressed far more RIPK3 than hepatocytes or HSCs (Figure 4G), indicating that they are likely the primary contributors to *RIPK3* upregulation observed in MASLD livers.

At the protein level, 148M macrophages exhibited increased phosphorylation of RIPK3 (Figure 4H) and downstream MLKL (Figure 4I), indicating an enhanced necroptotic signaling. This was further supported by a greater proportion of propidium iodide (PI)-positive cells (Supplemental Figure S3I, J, http://links.lww.com/HC9/C235) and increased membrane-associated phosphorylated MLKL (Figure 3J), hallmark features of necroptotic execution. Consequently, 148M macrophages released higher levels of danger-associated molecular patterns (DAMPs), IL-1α, and HMGB1 (Figure 4K), further supporting heightened necroptotic activity in 148M macrophages.

Together, these data suggest that the 148M variant enhances macrophage susceptibility to lipotoxicity-induced necroptosis, contributing to increased inflammation and cell death in the MASLD-like liver environment.

### NACC1 is a key upstream regulator of RIPK3 in macrophages

**FIGURE 5:**
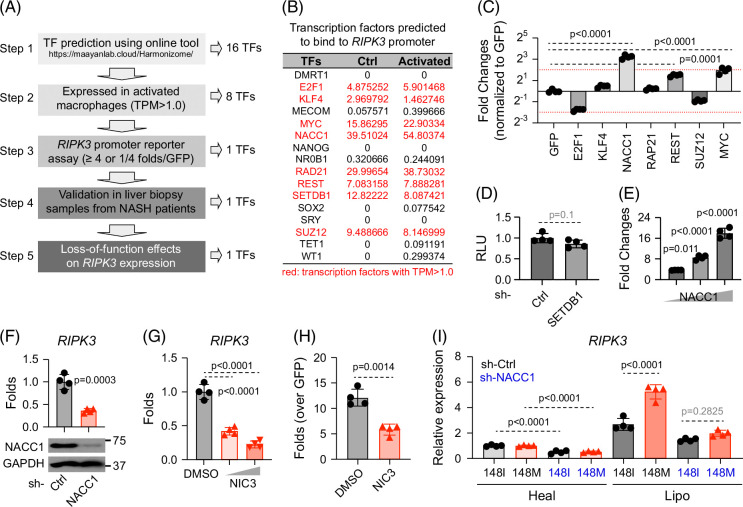
NACC1 is a key upstream regulator of RIPK3 in macrophages. (A–C) Schematic representation of the analysis flow to identify transcription factors that regulate *RIPK3* expression in macrophages. Predicted transcription factors were first filtered through expression in activated macrophages [cutoff for TPM is >1.0 (highlighted in red) (B)], then through functional testing using an authentic *RIPK3* promoter reporter in HEK293 cells (C). (D) Since the SETDB1 protein is too big, its effect on RIPK3 expression was examined by shRNA-mediated downregulation followed by reporter assay in HEK293 cells. (E) HEK293 cells were transfected with increasing amounts of pCAGGS-NACC1 (0.05, 0.2, 0.4 μg/well of 24-well plate), together with *RIPK3* reporter. At 40 hours post-transfection, cells were harvested for luciferase assay. (F, G) Human iPSC-derived macrophages were transduced with control shRNA or *NACC1* shRNA, or treated with NIC3 (15 μM and 30 μM). Cells were then harvested for analysis of *RIPK3* transcripts and western blot analysis (F). (H) HEK293 cells were transfected with pCAGGS-NACC1 and *RIPK3* luciferase reporter, treated with vehicle control DMSO or NIC3 (30 μM) for 30 hours before being harvested for luciferase assay. (I) Human iPSC-derived macrophages were transduced with control shRNA or *NACC1* shRNA, exposed to conditioned supernatants for 4 days, and then harvested for analysis of *RIPK3* transcripts. Shown are the means ± SD from n=4 independent experiments. Statistical analysis was performed using unpaired *t* tests with Welch correction or one-way ANOVA/Tukey post-hoc test to calculate exact *p* values. Abbreviations: iPSC, induced pluripotent stem cell; NACC1, nucleus accumbens associated 1; RIPK1/3, receptor-interacting serine/threonine kinase 1/3; TPM, transcripts per million.

To identify upstream regulators of RIPK3 in macrophages, we followed the workflow in Figure 5A. Using the Harmonizome database (https://maayanlab.cloud/Harmonizome/), we first compiled transcription factors predicted to bind the *RIPK3* promoter and narrowed this list to 8 candidates with robust expression in activated macrophages (Figure 5B).

To test their functional relevance, we employed a *RIPK3* promoter-driven luciferase reporter. Among the candidates, NACC1 (nucleus accumbens associated 1) emerged as the most potent activator, significantly enhancing luciferase activity compared with the GFP control (Figures 5C, D) and doing so in a dose-dependent manner (Figure 5E).

We then asked whether altering NACC1 levels would influence endogenous RIPK3 expression. *NACC1* knockdown markedly reduced RIPK3 levels in macrophages (Figure 5F). Treatment with NIC3, a small-molecule inhibitor of NACC1, similarly reduced *RIPK3* mRNA levels (Figure 5G) and NACC1-induced *RIPK3* promoter activity (Figure 5H). Consistently, *NACC1* downregulation reduced *RIPK3* expression under both healthy and lipotoxic conditions, and under lipotoxic stress largely eliminated the genotype-dependent increase in *RIPK3* (Figure 5I).

Together, these findings identify NACC1 as a key transcriptional regulator of RIPK3 in macrophages, particularly under conditions of lipotoxic stress relevant to MASLD pathogenesis.

### NACC1 downregulation attenuates necroptosis and genotype-dependent differences

**FIGURE 6:**
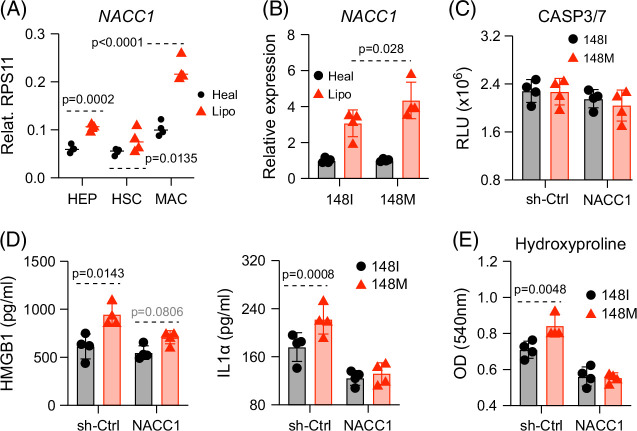
NACC1 downregulation attenuates necroptosis and genotype-dependent differences. (A) Liver cultures harboring all 148I cells were exposed to healthy or lipotoxic medium for 2 weeks. Individual cell types were purified for analysis of *NACC1* transcripts. (B) Liver cultures harboring either 148I or 148M macrophages were exposed to healthy or lipotoxic medium for 2 weeks, and macrophages were harvested for analysis of *NACC1* transcripts. (C–E) Liver cultures were assembled as described in Figure 1A, except for macrophages being transduced with control shRNA or *NACC1* shRNA. Liver cultures were then exposed to lipotoxic medium for 2 weeks before macrophages were analyzed for caspase 3/7 activity (C), supernatants for HMGB1 and IL-1α levels (D), and HSCs for hydroxyproline levels (E). Shown are the means ± SD from n=4 independent experiments. Statistical analysis was performed using unpaired *t* tests with the Welch correction or one-way ANOVA/Tukey post-hoc test to calculate exact *p* values. Abbreviations: HEP, hepatocytes; HMGB1, high mobility group box 1; HSC, hepatic stellate cells; IL1α, interleukin 1 alpha; MAC, macrophages; NACC1, nucleus accumbens associated 1.

To further examine the link between NACC1 and RIPK3 expression in macrophages, we first evaluated NACC1 expression across the major cell types in our liver culture. Under healthy conditions, NACC1 levels were similar in hepatocytes, macrophages, and HSCs. Following lipotoxic stress, however, NACC1 expression was upregulated in macrophages (Figure 6A), paralleling the pattern observed for RIPK3 (Figure 4G). A similar induction pattern was observed in primary human KCs and monocyte-derived macrophages treated with conditioned medium from lipotoxic liver cultures (Supplemental Figures S4A, B, http://links.lww.com/HC9/C235) and in KCs from the murine MASLD model described earlier (Supplemental Figure S4C, http://links.lww.com/HC9/C235).

Under lipotoxic conditions, 148M macrophages showed higher *NACC1* transcript levels than 148I cells (Figure 6B). As shown earlier, 148M macrophages accumulated higher levels of intracellular lipids and ROS under lipotoxic conditions (Supplemental Figures S1A, B, http://links.lww.com/HC9/C235, and Supplemental Figure 4A); hence, we asked whether these stress signals regulate NACC1. Because lipid overload and oxidative stress can activate the NF-κB pathway, we assessed NF-κB activity using a reporter assay. Both lipotoxic medium and H₂O₂-induced oxidative stress increased NF-κB–dependent activity (Supplemental Figure S4D, http://links.lww.com/HC9/C235), with a greater response in 148M macrophages (Supplemental Figure S4E, http://links.lww.com/HC9/C235). Consistent with this, canonical NF-κB target genes *ICAM1* and *CCL2* were elevated (Supplemental Figure S4F, http://links.lww.com/HC9/C235), which is in line with our previous work.^4^ To directly test the role of NF-κB signaling in regulating NACC1 expression, we knocked down *MYD88*, an upstream adapter of the NF-κB pathway in macrophages. MYD88 depletion reduced *NACC1* induction in both genotypes (Supplemental Figure S4G, http://links.lww.com/HC9/C235), demonstrating that NF-κB signaling contributes to *NACC1* upregulation during lipotoxic stress, with a heightened response in 148M macrophages.

Functionally, *NACC1* knockdown minimally affected macrophage apoptosis (Figure 6C), but nearly abolished genotype-dependent differences in necroptosis, as reflected by reduced secretion of IL1α and HMGB1 (Figure 6D). This suggests a specific role for NACC1 in regulating necroptotic, but not apoptotic, cell death in response to lipotoxic stress in macrophages.

Finally, in the multicellular liver culture system, macrophage *NACC1* knockdown eliminated the genotype-associated increase in hydroxyproline accumulation (Figure 6E), indicating that elevated NACC1 expression in 148M macrophages substantially contributes to the enhanced fibrotic response under lipotoxic conditions.

### Chemical inhibition of NACC1 using NIC3 protects against lipotoxic injury

Finally, we evaluated the therapeutic potential of the NACC1 inhibitor NIC3 in mitigating lipotoxic injury. Although the precise molecular mechanism of NIC3 remains incompletely defined, it is thought to impair NACC1 transcriptional activity, likely by disrupting its BTB/POZ domain-mediated interactions or DNA-binding capacity.^23^

**FIGURE 7:**
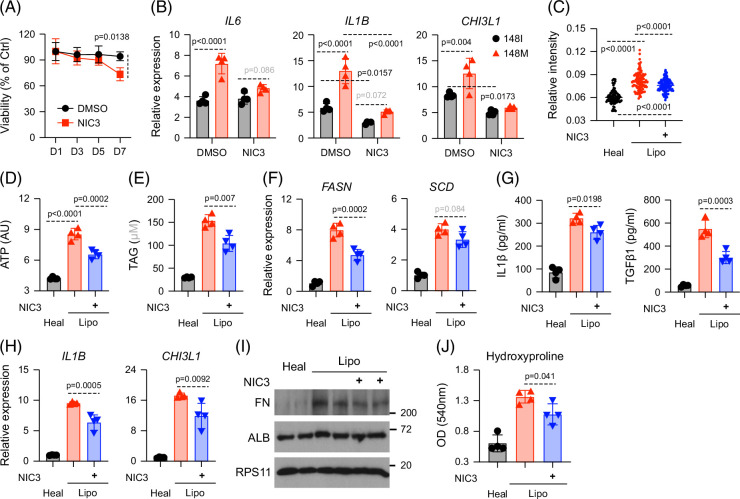
Chemical inhibition of NACC1 using NIC3 protects against lipotoxic injury. (A) Liver cultures containing all 148I cells were exposed to a lipotoxic medium with DMSO or NIC3 (30 μM). Following treatment, all cells were collected for the cell viability assay. (B) Liver cultures were assembled as described in Figure 1A and exposed to lipotoxic medium for 2 weeks before being analyzed by RT-qPCR. (C–G) Liver culture containing all 148I cells was exposed to healthy or lipotoxic medium for 2 weeks. Hepatocytes were analyzed for intracellular lipid content (C), intracellular ATP (D), triglyceride (TAG, E), and transcripts (F); supernatants for ELISA (G); macrophages for analysis of transcripts of inflammatory genes (H); hepatocytes and HSCs were analyzed by western blot (I); HSCs were purified for analysis of hydroxyproline levels. Shown are the means ± SD from n=4 independent experiments. Statistical analysis was performed using unpaired *t* tests with the Welch correction or one-way ANOVA/Tukey post-hoc test to calculate exact *p* values. Abbreviations: HSC, hepatic stellate cell; NACC1, nucleus accumbens associated 1; RT-qPCR, reverse transcription–quantitative polymerase chain reaction; TAG, triacylglycerol.

To assess safety, we first examined NIC3’s effects on cell viability. Short-term exposure to NIC3 (fewer than 5 days) had minimal impact, whereas longer treatments (>7 days) reduced cell viability (Figure 7A). Based on this, we administered NIC3 during the final 4 days of a 10-day lipotoxic treatment protocol.

NIC3 treatment substantially reduced the genotype-dependent inflammatory response in macrophages (Figure 7B). In 148I liver cultures, NIC3 modestly reduced intracellular lipid content (Figure 7C and Supplemental Figure S5A, http://links.lww.com/HC9/C235), ATP levels (Figure 7D), and TAG accumulation (Figure 7E), accompanied by downregulation of lipogenic enzymes (Figure 7F). NIC3 had minimal effects on genes involved in hepatocyte glucose metabolism (Supplemental Figure S5B, http://links.lww.com/HC9/C235), but reduced *CXCL8* expression (Supplemental Figure S5C, http://links.lww.com/HC9/C235).

At the system level, NIC3 lowered IL1β and TGFβ1 levels (Figure 7G). In macrophages, NIC3 similarly reduced pro-inflammatory gene expression (Figure 7H and Supplemental Figures S5D, E, http://links.lww.com/HC9/C235). In HSCs, NIC3 decreased fibronectin and collagen production (Figures 7I, J), indicating reduced fibrogenic activation.

Together, these findings suggest that pharmacologic inhibition of NACC1 with NIC3 attenuates PNPLA3-148M–driven inflammation and fibrosis, and may represent a promising strategy for preventing or treating MASLD.

## DISCUSSION

The 148M variant in *PNPLA3* is one of the most robust genetic risk factors for MASLD, yet its functional consequences in different liver cell types remain incompletely understood. While prior studies have predominantly focused on the role of PNPLA3 in hepatocytes and HSCs, our work identifies a novel and unexpected role for PNPLA3 in macrophages, particularly under lipotoxic stress. Using a genetically defined human iPSC-derived liver culture system, we demonstrate that macrophages harboring the 148M variant promote a more severe MASLD-like phenotype, characterized by enhanced inflammation, hepatocyte injury, and fibrotic activation.

A central finding of this study is that PNPLA3 expression is dynamically induced in macrophages under lipotoxic conditions, and that this upregulation occurs similarly in iPSC-derived macrophages, primary human KCs, monocyte-derived macrophages, as well as primary mouse KCs. Interestingly, although *PNPLA3* mRNA levels were comparable between 148I and 148M macrophages, protein levels were higher in the latter. This is likely due to post-transcriptional stabilization or reduced degradation of the 148M protein variant, similar to its regulation in hepatocytes and HSCs.^4^^,^^13^ Functionally, *PNPLA3* knockdown largely eliminated the heightened inflammatory phenotype in 148M macrophages, including IL-1β secretion, demonstrating that elevated PNPLA3 expression is a key driver of variant-specific inflammatory activation. A modest residual difference remained following *PNPLA3* knockdown, which likely reflects additional genotype-linked metabolic or stress-response pathways rather than PNPLA3 expression per se.

Mechanistically, we identified SREBF1 as a primary transcriptional driver of PNPLA3 induction in macrophages under lipotoxic stress. This observation aligns with previous studies linking SREBP1 activity to hepatic lipid metabolism and underscores the broader roles of this transcription factor in lipid-stressed immune cells.^18^ Notably, however, PNPLA3 expression was not completely abolished following *SREBF1* knockdown, indicating that additional transcriptional inputs contribute to its regulation in macrophages. The downstream consequences of PNPLA3-148M expression in macrophages included increased intracellular triacylglyceride and cholesterol accumulation, elevated LDH release, and notably, enhanced necroptotic cell death. Whereas apoptosis remained unchanged across genotypes, 148M macrophages demonstrated pronounced activation of the necroptotic pathway, characterized by increased expression and phosphorylation of RIPK3 and MLKL, greater plasma-membrane association of phosphorylated MLKL, and heightened membrane permeability. Consistent with necroptotic execution, 148M macrophages also released higher levels of DAMPs, including IL-1α and HMGB1, thereby amplifying inflammatory signaling within the liver microenvironment.

In our search for upstream regulators of RIPK3 expression, we identified NACC1 as a key transcription factor that displayed higher levels in 148M macrophages. Importantly, we show that NACC1 upregulation in 148M macrophages is driven by enhanced NF-κB signaling—likely triggered by intracellular lipid overload and oxidative stress. Elevated NACC1 expression was blunted by *MYD88* knockdown, establishing a mechanistic link between lipid stress, NF-κB activation, and NACC1 transcriptional upregulation.

Genetic knockdown or chemical inhibition of NACC1 effectively suppressed RIPK3 expression and diminished necroptosis without affecting apoptosis, thus directly targeting the pathologic cell death program. Importantly, NACC1 inhibition using the small-molecule compound NIC3 not only blunted macrophage inflammatory signaling but also conferred protection across the multicellular liver culture system. These findings collectively position NACC1 as a critical regulatory node linking PNPLA3 genotype to necroptotic macrophage death and inflammatory fibrosis in MASLD. While previous studies have implicated macrophages in the progression of MASLD and MASH,^24^ our study provides a direct mechanistic framework by which a common genetic variant modulates macrophage fate and function. Moreover, the demonstration that chemical inhibition of NACC1 can partially reverse these effects highlights its translational potential as a therapeutic target.

Collectively, our study expands the current understanding of PNPLA3-148M function by revealing its macrophage-specific impact on liver disease pathogenesis. We provide a mechanistic framework connecting genetic susceptibility, lipid-induced NF-κB activation, and NACC1-driven necroptotic cell death, with direct consequences for hepatic inflammation and fibrosis. Our study, therefore, uncovers new therapeutic opportunities for targeting inflammation and fibrosis in patients with PNPLA3-associated liver disease.

## Supplementary Material
