# Supplementary material for: A PNPLA3–NACC1–RIPK3 pathway mediates macrophage necroptosis and inflammation in MASLD
Source: Hepatol Commun. 2026 Jan 21;10(2):e0890. doi: 10.1097/HC9.0000000000000890 (PMC12826197; doi:10.1097/HC9.0000000000000890)
Supplement: Supplementary file 1 [file hc9-10-e0890-s001.docx]

**A PNPLA3–NACC1–RIPK3 Pathway Mediates Macrophage Necroptosis and Inflammation in MASLD**

Xinjia Wang, Lu Bian, Zhuoying Feng, Kyle O’Shaughnessy, Andrew C. Kwong, Eun Hee Ha, Lei Wang, Weibo Chen, and Xianfang Wu

**SUPPLEMENTAL DIGITAL CONTENT (SDC), MATERIALS & METHODS**

**Primary cell culture**. Primary human Kupffer cells were maintained in Lonza cell growth media according to the manufacturer’s instructions.

**Establishment of a multicellular liver culture**. To mimic healthy condition, BMM was supplemented with normal physiological levels of insulin (0.7nM) and glucose (6.0mM). To mimic lipotoxic condition, high levels of insulin (7.0nM), glucose (25.0mM) and free fatty acid (oleic acid, 68μM and palmitic acid, 45 μM) to mimic plasma concentrations of these factors in MASLD patients. In these experiments, half of the culture medium was replenished every two days.

**Separation of hepatocytes from hepatic stellate cells**. Isolation of hepatocytes from cocultures was performed as described previously ^4-6^. Briefly, hiPSC-derived co-cultures of hepatocytes and HSCs were washed once with Versene and then incubated in Versene at 37°C for 20–25 minutes to loosen cell–cell contacts. Cultures were subsequently washed with pre-warmed DMEM/F12 and incubated with a pre-warmed collagenase mixture (2.0 mg/ml collagenase, 1.0 mg/ml dispase, 100 U/ml DNase, 0.2% DMSO in HBM) at 37°C for 35–40 minutes. During this incubation, cells were gently pipetted to disrupt clumps and facilitate dissociation. Following digestion, cells were collected by adding Versene and centrifuged at 400 g for 5 minutes at room temperature. The pellet was resuspended in Versene and incubated again at 37°C for 45 minutes. By the end of this incubation, the majority of cells were dissociated into single cells. If necessary, the suspension was passed through a 100 µm cell strainer to remove residual clumps.

To separate hepatocytes from HSCs, single-cell suspensions were first blocked in 1% BSA and incubated on ice for 1 hour. Cells were then stained with mouse anti-ASGR1 antibody, followed by incubation with anti-mouse IgG microbeads, and subjected to magnetic separation at 4°C. Hepatocytes were collected from the magnetically bound fraction, while HSCs were obtained from the flow-through after an additional round of hepatocyte depletion. Purified individual cell types were collected for downstream analysis including transcriptomic analysis, immunofluorescent staining, western blot, and functional analyses.

**Experimental animals.** Male C57BL/6NTac mice previously fed the Gubra Amylin (GAN) MASH diet or a matched control diet for 18 weeks were obtained from Taconic Biosciences (model NASH-B8-M) and maintained on their assigned diets for an additional 10 weeks. Animals (three per cage) were housed in a barrier facility at 22 °C on a 12:12-hrs light–dark cycle.

For liver macrophage isolation, tissues were perfused via the portal vein at low pressure with an EDTA-containing buffer, then digested at 37 °C with collagenase type I and DNase I. The cell suspension was filtered through nylon mesh and centrifuged at 1,000 × g for 10 min, and non-parenchymal cells were enriched using Lymphocyte Separation Medium (MilliporeSigma, C-44010). After a PBS wash, cells were stained (20 min, protected from light) with anti-mouse F4/80 (clone T45-2342; BD Biosciences, 566787) and anti-mouse CD11b (clone M1/70; BD Biosciences, 561689), then sorted on a BD FACSAria™ II. Monocyte-derived macrophages (F4/80⁺CD11b^hi^) and Kupffer cells (F4/80^hi^CD11b⁺) were collected for downstream RNA extraction and RT-qPCR analysis. Post-sorting cells were F4/80⁺ (clone BM8; BioLegend, 123137) with >98% purity in all preparations. All procedures were conducted under protocols approved by the Cleveland Clinic Institutional Animal Care and Use Committee.

**Lentivector-based transduction**. Lentiviral particles-based shRNAs targeting genes of interest were cloned into the pLKO.1-puro vector using standard techniques. To generate lentiviral particles, packaging plasmids were transfected into Lenti-293T cells using Lipofectamine 2000 according to the manufacturer’s directions, medium was changed to DMEM supplement with 3% ESC-qualified FBS (Life Technologies) at 6 hr post transfection. Medium containing the lentiviral particles was harvested at 24 and 48 hrs post transfection and filtered through 0.2 μm filter. Subsequently, lentiviral particles were further concentrated (100:1) using Lenti-X Concentrator (Takara Bio) according to the manufacturer’s directions.

To transduce hiPSC-derived macrophages, hiPSC-derived CD34^+^ cells were exposed twice to a mixture of lentivectors and maintenance medium (1:10 ratio) in the presence of polybrene (4μg/ml) and a JAK inhibitor (Pyridone 6, 100nM), with each exposure lasting 4 hrs. Following transduction, thorough washing with pre-warmed DMEM/F12 was performed, and the cells were maintained in complete macrophage differentiation medium in the presence of 1μg/ml puromycin. CD163^+^ macrophages were further purified using CD163 MicroBead Kit (Miltenyi Biotec), according to the manufacturers’ instructions.

**Oil-red staining.** To stain lipid droplets in hiPSC-derived hepatocytes, purified cells were cultured on matrigel-coated plate in the respective medium for 6 to 8 hrs. Subsequently, the cells were fixed with 10% formalin for 30 minutes, followed by a 10-minute wash with 60% isopropanol at room temperature. Cells were then stained with 0.18% freshly prepared oil-red in PBS for 4 min at room temperature and washed thoroughly with double-distilled water four times. Nuclei were stained with DAPI for 1 min at room temperature. Images were captured using an Olympus IX73 Inverted Fluorescence Microscope System (Tokyo, Japan). Oil red quantification was performed as total red staining intensity at single cell level using ImageJ software.

To quantify lipid content at the single-cell level, we analyzed fluorescence microscopy images in which lipid droplets and nuclei were co-stained and captured in a single channel. Using ImageJ software, each image was first converted to grayscale, and nuclei were segmented using Otsu thresholding followed by morphological filtering and watershed-based separation to identify individual cells. We then measured the lipid signal intensity within each segmented nucleus, extracting key parameters including nuclear area, mean lipid intensity, and the range of lipid signal (maximum and minimum intensity) for downstream analysis using.

**Quantitative real-time RT-PCR (RT-qPCR)**. Total RNA was isolated from cell lysates using the RNAeasy Mini Kit (Qiagen, Germany) or PureLink RNA Mini Kit (Invitrogen) followed by reverse transcription using RevertAid First Strand cDNA Synthesis (Thermo Fisher). Gene expression was quantified using the AzuraView GreenFast qPCR Blue Mix (Azura Genomics) on a LightCycler 480 Instrument (Roche Life Science, Germany) or a QuantStudio3 Instrument (Applied Biosystems) with gene-specific primers.

PCR conditions were as follows: initial denaturation step at 50°C for 2 min and 95°C for 10 min, then 45 cycles of 95°C for 15 sec, 56°C for 15 sec, and 72°C for 20 sec; followed by a melting step of 95°C for 10s, 65°C for 10s and a 0.07°C/s decrease from 95°C; and finally, a cooling step of 50°C for 5s. PCR product specificity was confirmed by a melting-curve analysis. Fold changes in mRNA expression were determined using the ΔΔCt method relative to the values in control samples as indicated in figure legends, after normalization to housekeeping proteins (RPS11 or GAPDH). Results are presented as means ± standard deviation (SD), unless stated otherwise. Comparisons between groups/cells were made using the two-tailed t-test with Welch’s corrections or One-way ANOVA/Tukey’s post-hoc test to calculate exact p-values, unless stated otherwise. Statistical analysis was performed in Graph Pad PRISM 10.

**Western blot analysis**. Cells were directly lysed in 2x SDS lysis buffer (2mL Tris-HCl (pH 6.8), 50% glycerol, 10% SDS, 0.5% bromophenol blue, and freshly added 10% β-mercaptoethanol) and cell lysates were separated by 7.5%, 10%, or 12% sodium dodecyl sulfate-polyacrylamide gel electrophoresis, in MES or MOPS buffer, followed by transfer onto polyvinylidene fluoride (PVDF) membrane (EMD Millipore). For comparison between different groups or treatments, GAPDH, β-actin, or RPS11 were used as housekeeping protein controls, as indicated in the figures and figures legends.

**Measurement of intracellular ATP**. Purified hiPSC-derived hepatocytes and macrophages were washed with D-PBS twice and seeded into 96-well plate (3 x 10^4^/well). The intracellular ATP was measured using the CellTiter-Glo luminescent cell viability assay kit (Promega) according to the manufacturer’s instructions.

**Quantification of intracellular triglyceride (TAG)**. Purified hiPSC-derived hepatocytes and macrophages were washed with D-PBS twice before subject to cell lysis. The intracellular TAG was measured using the Triglyceride-Glo assay kit (Promega) according to the manufacturer’s instructions.

**Quantification of collagen**. Purified hiPSC-derived hepatic stellate cells or coculture of hepatocytes and hepatic stellate cells were subject to quantification using a colorimetric hydroxyproline assay kit (Millipore Sigma) according to the manufacturer’s instructions.

**ELISAs**. To measure the secreted levels of human cytokines, including pro-inflammatory cytokines (IL6, TNFα, HMGB1, IL1α, and IL1β) and profibrotic cytokines (TGFβ1) in the liver cultures or monoculture of macrophages under different experiment settings, supernatant was collected at indicated time points and cytokine levels were quantified by ELISA kits (R&D Systems), according to the manufacturers’ instructions.

**Quantification of intracellular total cholesterol**. Purified hiPSC-derived hepatocytes and macrophages were washed with D-PBS twice before subject to cell lysis. The intracellular total cholesterol was measured using the Cholesterol/Cholesterol Ester-Glo assay kit (Promega) according to the manufacturer’s instructions.

**Quantification of lactate dehydrogenase (LDH)**. LDH in cell culture supernatants were quantified using a colorimetric assay kit (ThermoFisher Scientific), according to the manufacturers’ instructions.

**Quantification of caspase 3/7 and caspase 1**. Caspase 3/7 and caspase 1 activity in purified hiPSC-derived macrophages were quantified by Caspase-Glo kits (Promega), according to the manufacturers’ instructions.

**Luciferase reporter assay**. HEK293 cells were transfected with GFP or individual transcription factor expression plasmid, together with RIPK3 promoter-driven Firefly luciferase reporter and a CMV promoter-driven Renilla luciferase plasmid. Luciferase assays were conducted utilizing the Dual-Luciferase Reporter Assay System (Promega) in a VarioSkan LUX Multi-Mode Plate Reader (Thermo Scientific) or BMG Labtech (Germany)

**Apoptosis assay**. Purified hiPSC-derived macrophages were stained with Annexin V-FITC dye (Cell Signaling Technologies) for 10 minutes in the dark on ice prior to flow cytometry analysis.

**Polysome profiling assay**. Macrophages were pretreated with cycloheximide (CHX; 50 µg/ml) for 5 min at 37°C to arrest ribosomes on mRNAs, washed once with ice-cold PBS supplemented with CHX (50 µg/ml), and lysed on ice in polysome lysis buffer (20 mM Tris-HCl pH 7.4, 150 mM NaCl, 5 mM MgCl₂, 1% Triton X-100, 1 mM DTT, 50 µg/ml CHX, RNase inhibitor, and protease inhibitors). Lysates were incubated on ice for 10 min with gentle mixing and clarified by centrifugation (14,000 × g, 10 min, 4°C). Equal amounts from each condition were layered onto pre-formed 10–50% sucrose gradients (in 20 mM Tris-HCl pH 7.4, 150 mM NaCl, 5 mM MgCl₂, 50 µg/mL CHX) and centrifuged in an SW41Ti rotor (36,000 rpm, 2 h, 4°C, no brake). Gradients were fractionated, with 0.5 ml per fraction, for RNA extraction (TRIzol LS, ThermoFisher). Purified RNA was reverse-transcribed and transcript abundance in each fraction was quantified by RT-qPCR.

**RNA stability assay**. *PNPLA3* transcript stability in macrophages was quantified by transcriptional shutoff using actinomycin D. Cells were treated with actinomycin D (5 μg/ml) and harvested at 0, 2, 4, 6, and 10 hours. At each timepoint, cells were washed with ice-cold PBS and lysed in lysis buffer. Total RNA was extracted, treated with DNase I to remove genomic DNA contamination, and reverse-transcribed using random hexamers to ensure uniform coverage across transcript regions. *PNPLA3* transcripts were then quantified by RT-qPCR. Normalized transcript abundance was expressed relative to the t = 0 timepoint.

**Protein stability assay**. PNPLA3 protein stability in macrophages was assessed using a cycloheximide (CHX) chase assay. Cells were treated with CHX (50 µg/mL) to inhibit *de novo* protein synthesis and harvested at 0, 0.5, 1, 2, 4, and 6 hrs post-treatment. Cell lysates were prepared in RIPA (50 mM Tris-HCl pH 7.4, 150 mM NaCl, 1% NP-40, 0.5% sodium deoxycholate, 0.1% SDS) supplemented with protease inhibitors, and equal protein amounts were analyzed by western blot.

**Subcellular Protein Fractionation**. Fractionation of human iPSC-derived macrophages was done using a commercial kit (ThermoFisher Scientific), according to the manufacturers’ instructions. EGFR was used as a positive control for membrane fraction.

**Statistical Analysis**. Detailed information regarding all statistical tests, the specific value of 'n' and its representation (e.g., number of cell clones or experimental replicates) can be found in the respective figure legends. Graphs depict results presented as means ± standard deviation (SD). Group comparisons were conducted using the Unpaired t test with Welch’s correction or One-way ANOVA/Tukey's post-hoc test, as appropriate, to calculate precise p-values unless otherwise specified. Statistical analyses were carried out using Graph Pad PRISM 10. All p-values are reported, and a p-value greater than 0.05 denotes statistical non-significance.

**DATA AVAILABILITY STATEMENT**

Upon publication, the research data (including raw and processed data and detailed methods) associated with this manuscript, and the processed data of previously published RNA-sequencing data (GSE135251 and GSE192742) used for transcriptomic analysis and validation will be freely available to research community.

**SUPPLEMENTAL DIGITAL CONTENT (SDC), FIGURES**

**Figure 1**

**
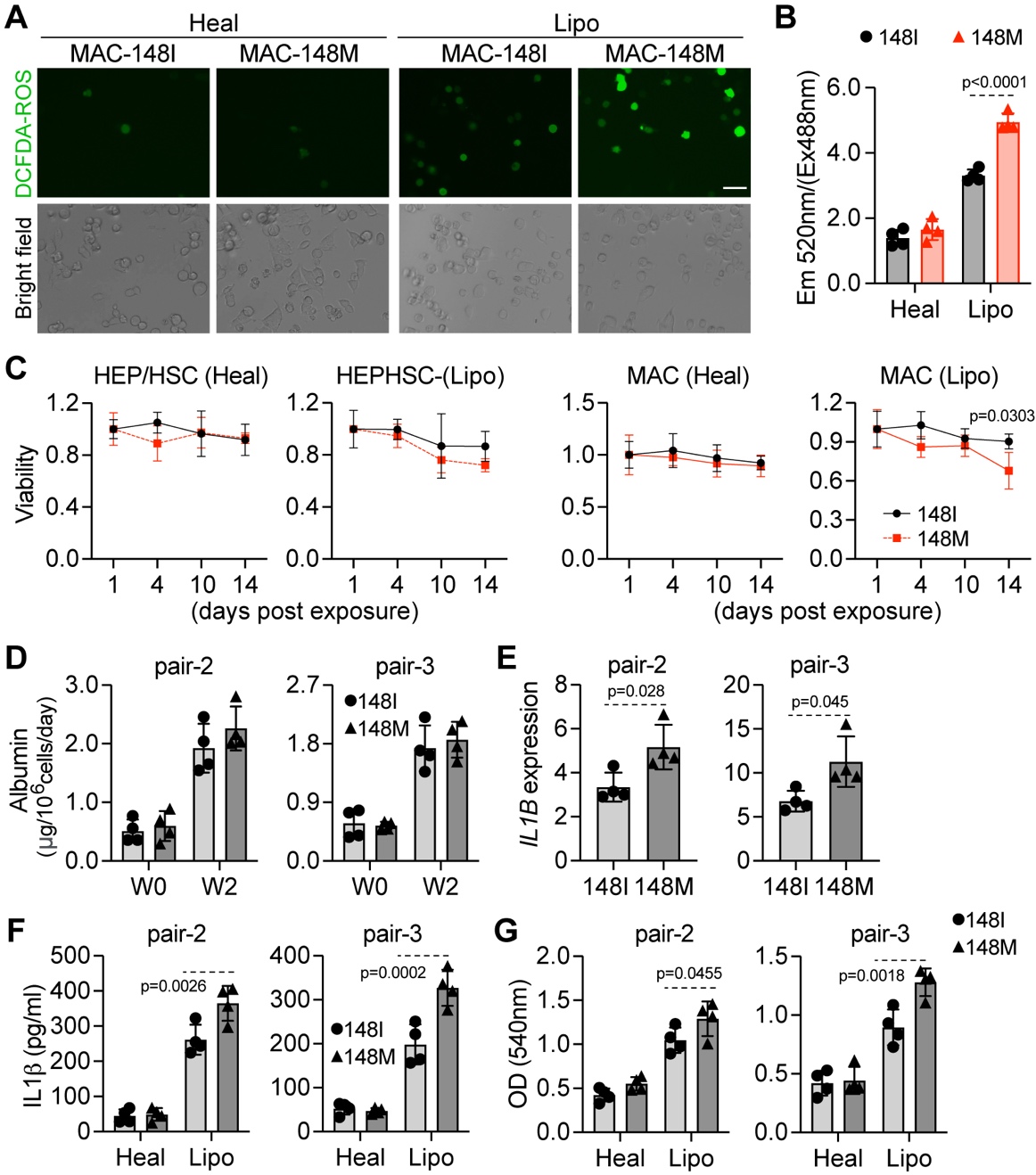
**

**Figure S1. Macrophage-specific PNPLA3-148M variant promotes inflammatory and fibrotic phenotypes.**

Liver cultures harboring either 148I or 148M macrophages were exposed to healthy or lipotoxic medium for 2 weeks.

(**A-B**). Macrophages were harvested for analysis of reactive oxygen species by DCFDA assay (A) and quantified for green fluorescent signals (B). Representative images were shown (scale bar: 100μm); Shown are mean ± SD from n=4 independent experiments.

(**C**). At the indicated time points, cells were collected for analysis of cell viability. Data were normalized to day 1. Shown are mean ± SD from n=4 independent experiments.

(**D**). Different pairs of isogenic liver cultures harboring either 148I or 148M macrophages, as described in figure 1A, were cultured in healthy milium for 2 week. At the indicated time points, cultures were subject to analysis of albumin secretion by ELISA. Shown are mean ± SD from n=4 independent experiments.

(**E-G**). Different pairs of isogenic liver cultures harboring either 148I or 148M macrophages, as described in figure 1A, were exposed to healthy or lipotoxic medium for 2 weeks. Macrophages were collected for analysis of *IL1B* transcript by RT-qPCR (E); supernatants were harvested for analysis of secreted IL1β by ELISA (F); and HSCs were purified for analysis of collagen levels by the hydroxyproline assay (G). Shown are mean ± SD from 4 independent experiments.

Statistical analysis was performed using Unpaired t tests with Welch’s correction or One-way ANOVA/Tukey’s post-hoc test to calculate exact p-values.

**Figure S2**

**
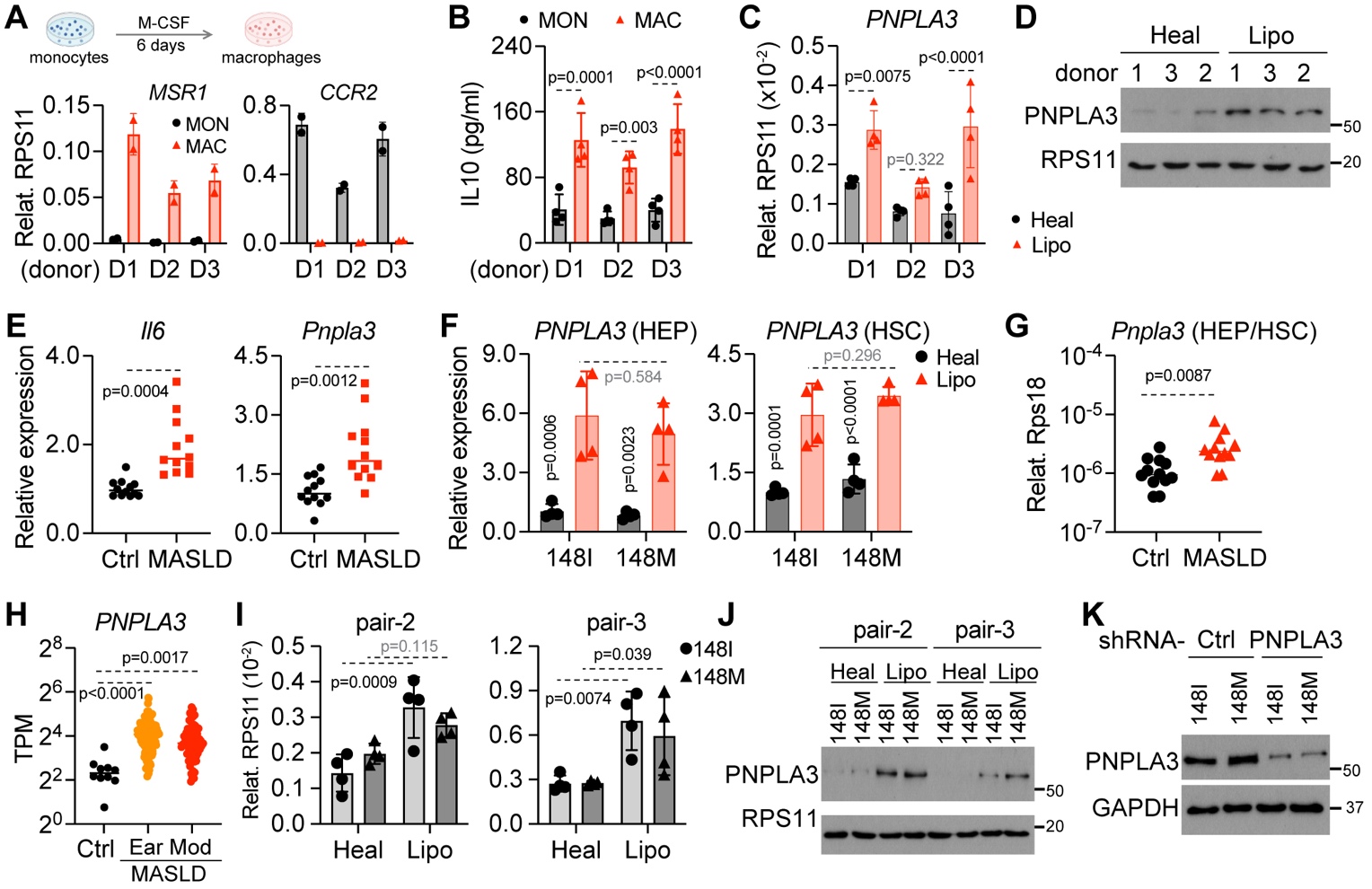
**

**Figure S2. PNPLA3 is upregulated in macrophages under lipotoxic stress.**

(**A**). Primary human monocytes (from three different donors) were differentiated into macrophages using M-CSF. Monocytes and differentiated macrophages were collected for analysis of transcript levels of macrophages marker *MSR1* and monocyte marker *CCR2* by RT-qPCR. Shown are mean ± SD from n=4 independent experiments.

(**B**). From the experiment described in (A), monocytes and differentiated macrophages were exposed to LPS (500ng/ml) for 2 days before the supernatants were collected for analysis of secreted IL10 by ELISA. Shown are mean ± SD from n=4 independent experiments.

(**C-D**). From the experiment described in (A), differentiated macrophages were exposed to conditioned medium collected from liver cultures at 2 weeks post treatment with the indicated medium for 5 days before being analyzed for *PNPLA3* transcript levels by RT-qPCR (C) or analyzed by western blot of indicated proteins (D). RPS11 was used as a loading control. Shown in (C) are mean ± SD from n=4 independent experiments.

(**E**). Macrophages (F4/80⁺CD11b^+^) were sorted from six MASLD mice (MASH-B6-M) and six control mice (MASH-B6-CONTRAL-M) at 28 weeks of feeding and subject to analysis of *Il6* and *Pnpla3* transcript levels by RT-qPCR, with technical duplicates for each mouse.

(**F**). Liver cultures were assembled as described in figure 1A and exposed to the indicated culture medium for 2 weeks. Hepatocytes (left) and HSCs (right) were purified for analysis of *PNPLA3* transcript levels by RT-qPCR. Values are normalized to cells from 148I cultures under healthy condition. Shown are mean ± SD from n=4 independent experiments.

(**G**). Hepatocytes (containing a small amount of HSCs) were collected from six MASLD mice (MASH-B6-M) and six control mice (MASH-B6-CONTRAL-M) at 28 weeks of feeding and subject to analysis of *Pnpla3* levels by RT-qPCR, with technical duplicates for each mouse. Data were normalized to housekeeping genes Rps18.

(**H**). Transcript levels of PNPLA3 were analyzed using transcriptomic data from MASLD patients (GSE135251). Patients were divided according to their disease stage: early (Ear) or moderate (Mod).

(**I-J**). As described in figure S1D, different pairs of isogenic liver cultures harboring either 148I or 148M macrophages were exposed to healthy or lipotoxic medium for 2 weeks. Macrophages were collected for analysis of *PNPLA3* transcript by RT-qPCR (I) or by western blot analysis of indicated proteins (J). RPS11 was used as a loading control. Shown in (I) are mean ± SD from n=4 independent experiments.

(**K**). Liver cultures were assembled as described in figure 1A, except for macrophages being transduced with control shRNA or shRNA against *PNPLA3*. Liver cultures were then exposed to lipotoxic medium for 2 weeks before macrophages were harvested for western blot analysis of PNPLA3 and housekeeping protein GAPDH, which serves as a loading control.

Statistical analysis was performed using Unpaired t tests with Welch’s correction or One-way ANOVA/Tukey’s post-hoc test to calculate exact p-values.

**Figure S3**

**
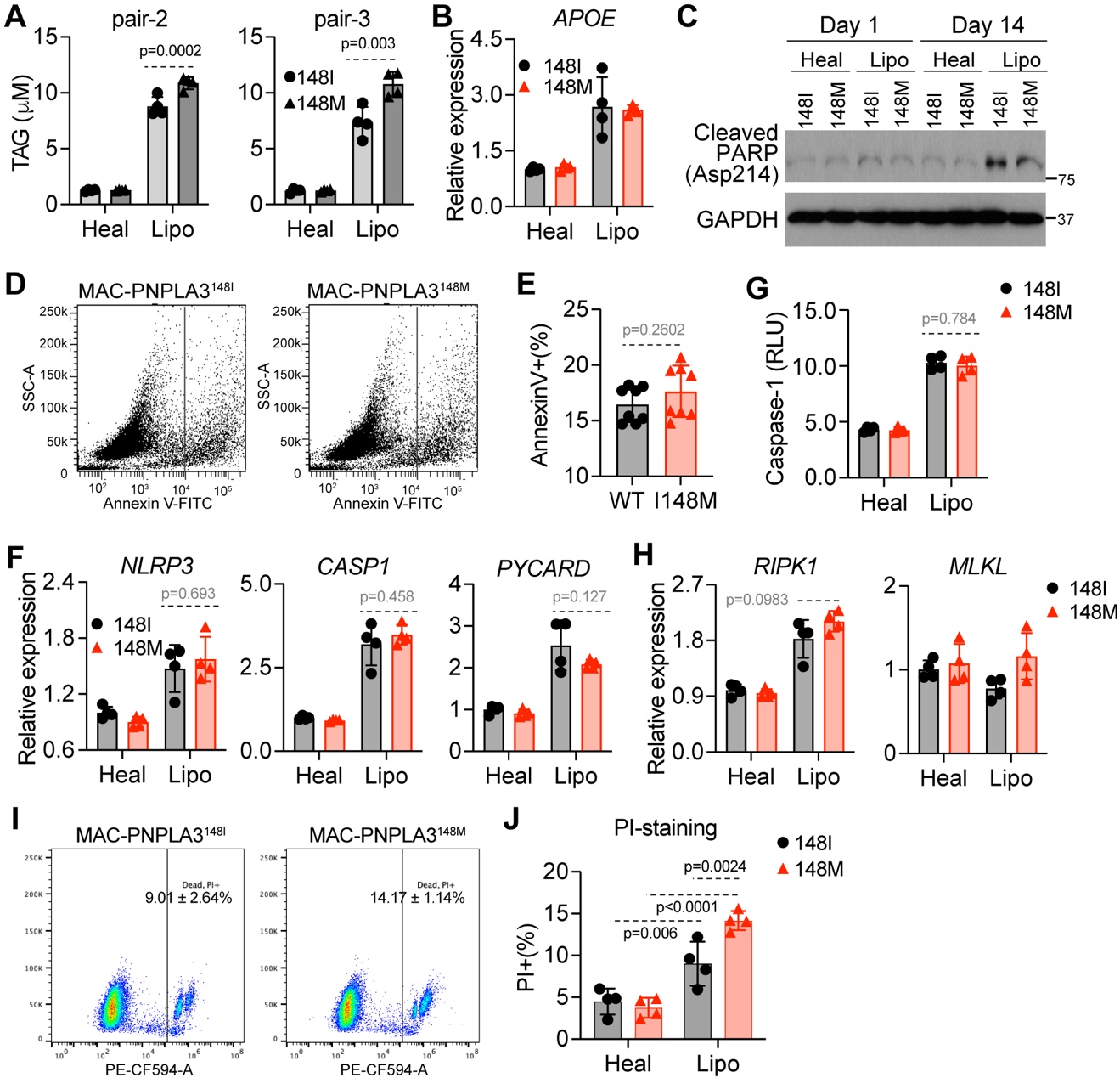
**

**Figure S3. 148M macrophages exhibit enhanced lipid accumulation, LDH release, and necroptosis, independent of apoptosis or pyroptosis.**

(**A**). As described in figure S1D, different pairs of isogenic liver cultures harboring either 148I or 148M macrophages were exposed to healthy or lipotoxic medium for 2 weeks. Macrophages were collected for analysis of intracellular TAG levels. Shown in are mean ± SD from n=4 independent experiments.

Liver cultures harboring either 148I or 148M macrophages were exposed to healthy or lipotoxic medium for 2 weeks.

(**B**). Macrophages were collected for analysis of transcript levels of *APOE* by RT-qPCR. Shown are mean ± SD from n=4 independent experiments.

(**C**). Macrophages were harvested at the indicated time points for western analysis of cleaved PARP. GAPDH serves as a loading control.

(**D-E**). Representative images showing flow cytometry analysis of Annexin V positive cells of macrophages harvested at 13 days post treatment (D). Summary of 4 independent replicates were shown in (E).

(**F, H**). Macrophages were harvested for analysis of transcript levels of inflammasome component genes (F) and necroptosis component genes (H). Shown are mean ± SD from n=4 independent experiments.

(**G**). Macrophages were harvested for analysis of caspase 1 activity. Shown are mean ± SD from n=4 independent experiments.

(**I-J**). Macrophages were collected for flow cytometry analysis of PI-positive cells. Representative images of flow cytometry analysis were shown (I) and summary of 4 independent replicates were shown in (J).

Statistical analysis was performed using Unpaired t tests with Welch’s correction or One-way ANOVA/Tukey’s post-hoc test to calculate exact p-values.

**Figure S4**

**
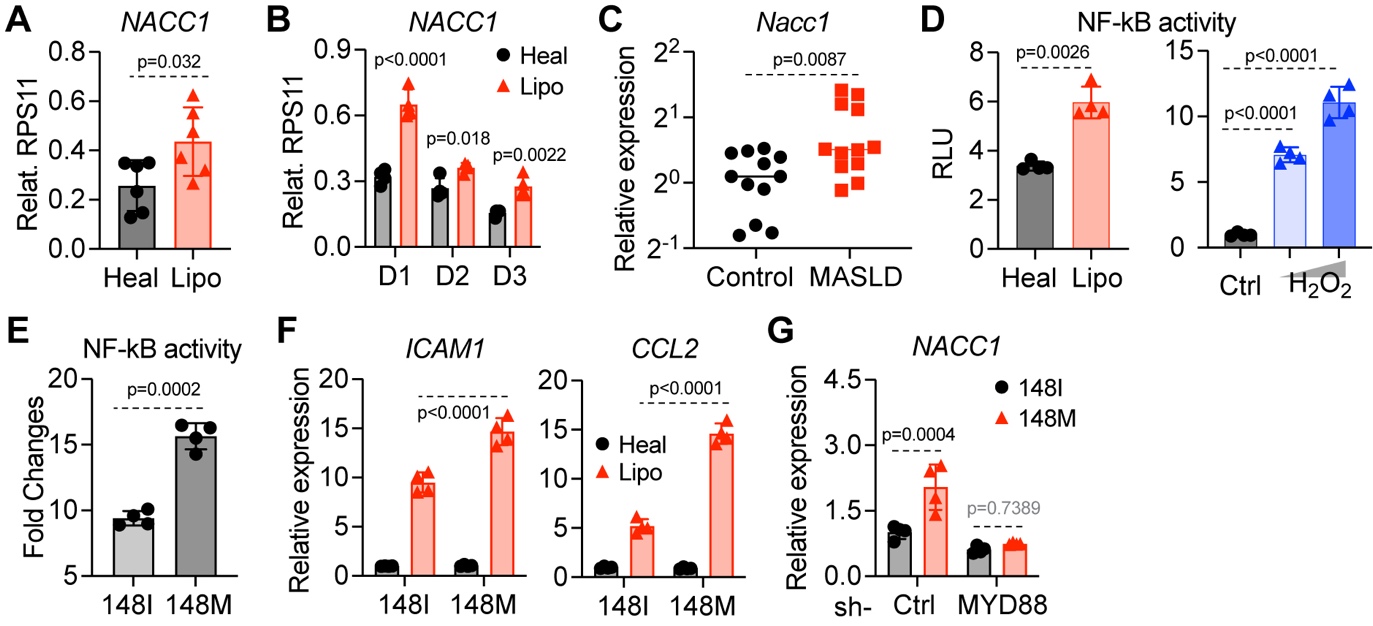
**

**Figure S4. NACC1 downregulation attenuates necroptosis and genotype-dependent differences.**

(**A**). Primary human Kupffer cells from three different donors were exposed to conditioned medium collected from liver cultures at 2 weeks post treatment with the indicated medium for 5 days before being analyzed for *NACC1* transcript levels by RT-qPCR, with technical duplicates for each donor.

(**B**). From the experiment described in figure S2A, monocyte-derived macrophages were exposed to conditioned medium collected from liver cultures at 2 weeks post treatment with the indicated medium for 5 days before being analyzed for *NACC1* transcript levels by RT-qPCR. Shown are mean ± SD from n=4 independent experiments for three different donors.

(**C**). Macrophages (F4/80⁺CD11b^+^) were sorted from six MASLD mice (MASH-B6-M) and six control mice (MASH-B6-CONTRAL-M) at 28 weeks of feeding and subject to analysis of *Nacc1* transcript levels by RT-qPCR, with technical duplicates for each mouse.

(**D**). 148I macrophages were transduced with a lentiviral-based NF-kB luciferase reporter before being treated with either conditioned medium from liver cultures for 3 days or H_2_O_2_ (50 μM) for 6 hrs (harvested at 24 hrs). Cells were then collected for luciferase assay. Shown are mean ± SD from n=4 independent experiments.

(**E**). 148I and 148M macrophages were transduced with a lentivector-based NF-kB luciferase reporter before being incorporated into liver cultures and then collected for luciferase assay at two weeks post lipotoxic medium exposure. Shown are mean ± SD from n=4 independent experiments.

(**F**). Liver cultures harboring either 148I or 148M macrophages were exposed to healthy or lipotoxic medium for 2 weeks and macrophages were harvested for analysis of transcript levels of *ICAM1* and *CCL2* by RT-qPCR. Shown are mean ± SD from n=4 independent experiments.

(**G**). Liver cultures harboring either 148I or 148M macrophages were exposed to healthy or lipotoxic medium for 2 weeks. At 4 days prior to harvesting cells, macrophages were transduced with control shRNA or shRNA against *MYD88*. Macrophages were then harvested for analysis of *NACC1* transcript levels by RT-qPCR. Shown are mean ± SD from n=4 independent experiments.

Statistical analysis was performed using Unpaired t tests with Welch’s correction or One-way ANOVA/Tukey’s post-hoc test to calculate exact p-values.

**Figure S5**

**
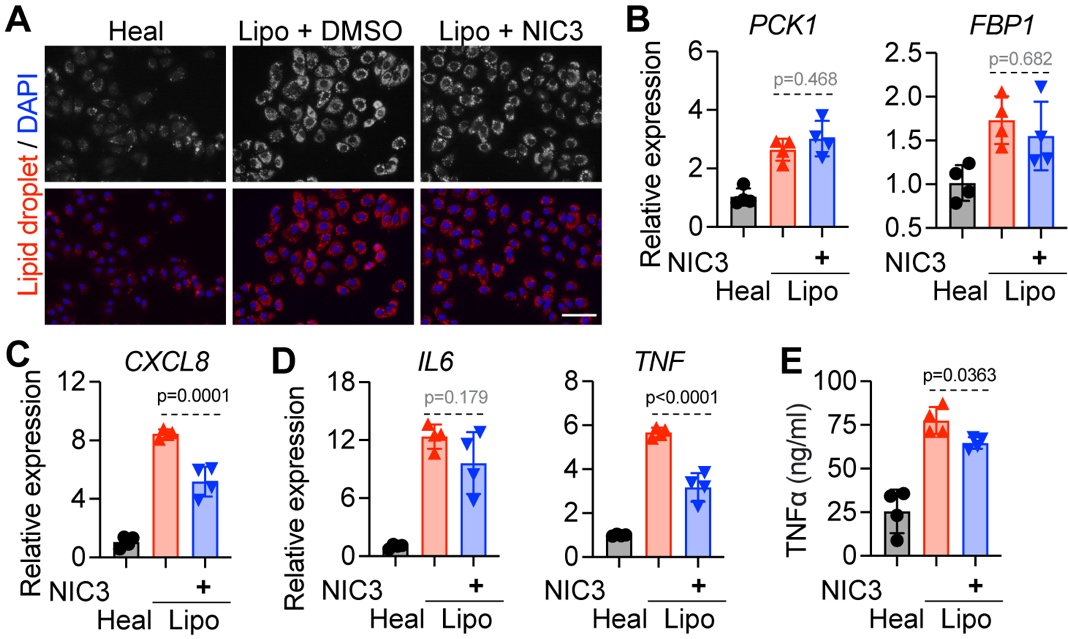
**

**Figure S5. Chemical inhibition of NACC1 using NIC3 protects against lipotoxic injury.**

(**A**). Liver cultures were exposed to a lipotoxic medium in the presence of either vehicle control DMSO or NIC3 (30 μM) for 2 weeks. Following treatment, hepatocytes were purified and re-seeded for quantification of lipid content by oil-red staining. Representative images are shown (scale bar: 100μm).

(**B-C**). In the experiments described in (A), following treatment, hepatocytes were purified for analysis of transcript levels of glucose metabolism-related genes *PCK1* and *FBP1* (B) and inflammatory cytokine gene *CXCL8* (C). Shown are mean ± SD from n=4 independent experiments.

(**D**).In the experiments described in (A), macrophages were harvested for analysis of transcript levels of inflammatory cytokine genes *IL6* and *TNF*. Shown are mean ± SD from n=4 independent experiments.

(**E**). In the experiments described in (A), supernatants were collected for analysis of inflammatory cytokine TNFα by ELISA. Shown are mean ± SD from n=4 independent experiments.

Statistical analysis was performed using One-way ANOVA/Tukey’s post-hoc test to calculate exact p-values.
